# The Need to Decide If All Estrogens Are Intrinsically Similar

**DOI:** 10.1289/ehp.7028

**Published:** 2004-05-19

**Authors:** Jonathan G. Moggs, John Ashby, Helen Tinwell, Fei Ling Lim, David J. Moore, Ian Kimber, George Orphanides

**Affiliations:** Syngenta CTL, Alderley Park, Cheshire, United Kingdom

**Keywords:** diethylstilbestrol, estrogen, gene expression, genistein, microarray, phytoestrogen, toxicogenomics, uterus

## Abstract

We used gene expression profiling to investigate whether the molecular effects induced by estrogens of different provenance are intrinsically similar. In this article we show that the physiologic estrogen 17β-estradiol, the phytoestrogen genistein, and the synthetic estrogen diethylstilbestrol alter the expression of the same 179 genes in the intact immature mouse uterus under conditions where each chemical has produced an equivalent gravimetric and histologic uterotrophic effect, using the standard 3-day assay protocol. Data are also presented indicating the limitations associated with comparison of gene expression profiles for different chemicals at times before the uterotrophic effects are fully realized. We conclude that the case has yet to be made for regarding synthetic estrogens as presenting a unique human hazard compared with phytoestrogens and physiologic estrogens.

The question of whether phytoestrogens and synthetic estrogens are toxicologically similar, or intrinsically different, presents a challenge to all involved in human hazard and risk assessments. Although there is a general concern that exposure to nanogram or microgram amounts of environmental estrogens may be associated with adverse health effects, in the public mind there is a widespread belief that foods and dietary supplements containing milligram quantities of phytoestrogens confer only health benefits. An implicit distinction therefore seems to have been drawn between synthetic and plant-derived estrogens—a belief sustained in the public mind by the assumption that natural is good and synthetic is bad—but an untested and potentially misleading notion for those involved with science-based human hazard/risk assessments.

Phytoestrogens and synthetic estrogens are generally considered separately in the literature. For example, Howdeshell et al. (1999) suggested a possible association between the advance in first estrus observed in mice exposed *in utero* to 2.4 μg/kg of the synthetic environmental estrogen bisphenol A and reports of an increased incidence of hypospadias in boys (Paulozzi et al. 1997) and the earlier sexual maturation of girls (Herman-Giddens et al. 1997)—the implication being that synthetic estrogens present a greater hazard than the much higher levels of phytoestrogens being consumed by those same children. In contrast, there are reports of an increased incidence of hypospadias in boys born to vegetarians (North and Golding 2000), of alterations in the menstrual cycle (Cassidy et al. 1994), and of reduced breast cancer incidences (Messina 1999) among women eating diets rich in phytoestrogens. Support for these epidemiologic observations comes from experimental studies indicating that advances in sexual development in rodents can be induced by their exposure to phytoestrogens (Casanova et al. 1999; Cassidy and Faughnan 2000; Safe et al. 2002). In contrast to these separate lines of inquiry, Newbold and colleagues have evaluated potential similarities between natural and synthetic estrogens. In seminal studies, they demonstrated that neonatal exposure of female mice to equipotent uterotrophic doses of the phytoestrogen genistein (GEN; Figure 1) or the synthetic estrogen diethylstilbestrol (DES) leads to an identical incidence of uterine adenomas at 18 months of age (Newbold et al. 2001). However, in attempting to draw parallels, or distinctions, between phytoestrogens and synthetic estrogens, it is imperative to consider growing awareness of the complexity of estrogen signaling pathway and the pleuripotential biologic activities of most organic chemicals—irrespective of their origin.

Estrogen signaling in mammalian cells is primarily mediated at the molecular level by two members of the nuclear receptor superfamily—estrogen receptors alpha (ER-α) and beta (ER-β). Ligand-activated ER-α and ER-β function as transcription factors, in conjunction with numerous coregulatory proteins, in order to activate or repress the transcription of ER-responsive genes (Hall et al. 2001; Moggs and Orphanides 2001). There is considerable variation in the binding affinity of ER-α and ER-β among different estrogens (Kuiper et al. 1998). In the case of the chemicals studied here, the physiologic estrogen 17β-estradiol (E_2_) and DES bind with a similar affinity to ER-α and ER-β, whereas GEN binds with approximately 20-fold higher affinity to ER-β than to ER-β (Kuiper et al. 1998). Concerning nonhormonal properties of the test chemicals (most of which have only be defined *in vitro*), GEN inhibits a range of enzymes, including tyrosine kinases (Akiyama et al. 1987), nitric oxide synthase (Duarte et al. 1997), and topoisomerase II (Okura et al. 1988), and also decreases calcium-channel activity (Potier and Rovira 1999), lipid peroxidation (Arora et al. 1998), and diacylglycerol synthesis (Dean et al. 1989). Likewise, DES is reported to induce aneuploidy in mammalian cells (Aardema et al. 1998) and to bind to rat liver DNA (Williams et al. 1993). More recently, some phytoestrogens were reported to inhibit the aromatase-mediated conversion of testosterone to E_2_ *in vitro* (Almstrup et al. 2002), and equol, the major circulating estrogenic metabolite associated with the dietary ingestion of phytoestrogens, is reported to selectively sequester dihydrotestosterone and thereby to act as a functional antiandrogen *in vivo* (Lund et al. 2004).

In order to advance understanding in this area, we decided to compare the genes expressed in the immature mouse uterus when it had grown in response to treatment with the estrogens E_2_, DES, and GEN. The immature mouse uterus was selected for our analysis because it is a major estrogen-responsive organ and forms the basis for a reference assay of estrogenic activity (Owens and Ashby 2002), including carcinogenesis (Newbold et al. 2001). Furthermore, it expresses both ER-α and ER-β (Weihua et al. 2000) and the androgen receptor (Frasor et al. 2003). We initially conducted a global analysis of gene expression in the mouse uterus at 1, 2, 4, 8, 24, 48, and 72 hr after exposure to a single high dose of either GEN (250 mg/kg) or E_2_ (400 μg/kg). These single high doses yielded a sustained uterotrophic response over 72 hr (Figure 2A) and were selected to avoid the complex transcriptional program that may result from the standard uterotrophic assay exposure regime in which each test compound is dosed by repeated administration on 3 consecutive days (Odum et al. 1997). Groups of 10 sexually immature mice [Alpk:APfCD-1; 19/20 days of age; maintained on RM1 diet (Special Diets Services Ltd., Witham, Essex, UK)] received a single subcutaneous injection of each compound or the test vehicle [arachis oil (AO); 5 mL/kg], and uterine RNA was isolated and pooled by group at each of the seven time points to determine gene expression levels among the 12,488 mouse genes represented on the Affymetrix MG-U74Av2 GeneChip (Affymetrix, High Wycombe, UK). Transcript profiling was performed using MG-U74Av2 GeneChip and Microarray Analysis Suite 5.0 (Affymetrix). Normalization and hierarchical clustering were performed with GeneSpring 6.0 (Silicon Genetics, Redwood City, CA, USA). MIAME (*Minimum Information About a Microarray Experiment*)-compliant microarray data are available as supplementary information and submitted to the Gene Expression Omnibus (GEO) database (GEO 2004). These data were analyzed using unsupervised hierarchical clustering and yielded temporal relationships between the expression profiles of 3,450 genes that were either up- or down-regulated (> 1.5-fold) by E_2_ and/or GEN (Figure 2B). Each chemical induced a similar, multistage transcriptional response (Figure 2B), although it is noteworthy that we observed variations in the magnitude and timing of both early (e.g., c-*fos*) and late (e.g., lactotransferrin) ER-responsive genes during the uterotrophic responses induced by E_2_ and GEN (Figure 2C).

A detailed description of the molecular functions of the genes affected, together with their association with physiologic changes during uterine growth, has been reported (Orphanides et al. 2003) and will be described in more detail in a future publication (Moggs et al., unpublished data).

These observations suggest that GEN does not induce “off-target” ER-independent transcriptional responses, that is, those associated with the properties of GEN other than estrogenicity. Furthermore, there was no evidence for the topoisomerase II–inhibiting properties of GEN in the bone marrow of the present mice despite demonstration of the sensitivity of that tissue to the potent micronucleus-inducing activity of the topoisomerase II inhibitor etoposide (data not shown). Together, these data led us to question whether a synthetic estrogen such as DES would also induce similar transcriptional responses in the immature mouse uterus.

In order to avoid temporal vagaries in gene expression (e.g., Figure 2C), we decided to anchor our transcript profiling data to the phenotype of the grown uterus by employing equipotent uterotrophic doses of E_2_, GEN, and DES. We compared the global gene expression profiles in the uteri of intact immature mice stimulated with three daily low doses of either GEN, DES, or E_2_, with an exposure regimen the same as that used in a standard 3-day uterotrophic assay (Odum et al. 1997). The route of administration and the doses of GEN and DES used were as described by Newbold et al. (2001) in their equivalent-outcome carcinogenicity bioassays of these two chemicals. Three independent replicates of four groups of sexually immature mice (Alpk:APfCD-1; 19/20 days of age; maintained on RM1 diet) received three daily subcutaneous injections of GEN (50 mg/kg), E_2_ (2.5 μg/kg), or DES (2 μg/kg). Control animals received the vehicle, AO (5 mL/kg). These doses elicited similar uterotrophic responses (72 hr after the initial dose; Figure 3A, Table 1) and identical histologic changes in the uteri of the treated animals (Table 1). Uterine RNA was isolated and pooled for each of the 12 groups and analyzed for changes in gene expression levels using the same Affymetrix microarray of 12,488 mouse genes. The data were analyzed using two independent statistical methods. First, unsupervised hierarchical clustering defined the global relationships (Euclidean distances) between the 12 gene expression profiles (Figure 3B). The three control groups clustered under one node, whereas the chemical treatment groups formed a separate node of compound-independent clusters, indicating equal similarity within and between the transcriptional responses induced by the three estrogens (Figure 3B). One-way analysis of variance (ANOVA), with Bonferroni (Holm 1979) correction (familywise error rate < 0.05) to minimize false positives, identified 179 genes where expression levels were altered by one or more chemical treatments (Figure 3C). Remarkably, Tukey post hoc testing revealed that all of these genes were affected in all nine compound treatment groups.

Table 2 highlights the high degree of similarity between the transcriptional responses to each of the three estrogens. These include established estrogen-responsive genes such as lactotransferrin, complement component 3, c*-fos*, small proline-rich protein 2A, and keratoepithelin (Hewitt et al. 2003; Naciff et al. 2003), together with many genes that have not previously been associated with estrogenicity (Table 2).

Although these three estrogens can alter the expression of some genes with different magnitudes [e.g., peptidyl arginine deiminase II is up-regulated to a lesser extent by E_2_ (1.86-fold ± 0.27) relative to GEN (9.11-fold ± 0.33) and DES (5.15-fold ± 1.53); Table 2], the present data show that the same genes are affected during equivalent uterotrophic responses. Previous studies have revealed both similarities and differences between transcriptional responses induced at a single time point after exposure to E_2_ and DES in the uteri of immature ovariectomized mice (Watanabe et al. 2003) and after exposure to either GEN, bisphenol A, or 17α-ethynyl estradiol in the reproductive tract of intact adult rats (Naciff et al. 2002). We suggest that these reported differences most probably arise from dose-dependent variations in the magnitude and kinetics of gene expression (Figure 2C), rather than from the operation of distinct mechanisms of estrogenic action.

Our data indicate that estrogens of differing provenance may have in common the potential for both beneficial and adverse health effects. This highlights the need for an holistic approach to hazard assessment wherein preconceptions are replaced by an objective assessment of the likely perturbations of physiologic functions caused by combined exposures to physiologic, synthetic, and plant-derived estrogens. This need is reinforced by data showing that plasma concentrations of isoflavones in infants fed soy formula are approximately 200 times higher than for those fed human milk (Setchell et al. 1997), by the estimated daily intake of approximately 29 mg of phytoestrogens for individuals taking dietary supplements (Committee on Toxicity of Chemicals in Food, Consumer Products and the Environment 2003), and by the demonstration that estrogens of different provenance can act additively in the rodent uterus (Tinwell and Ashby 2004).

## Figures and Tables

**Figure 1:**
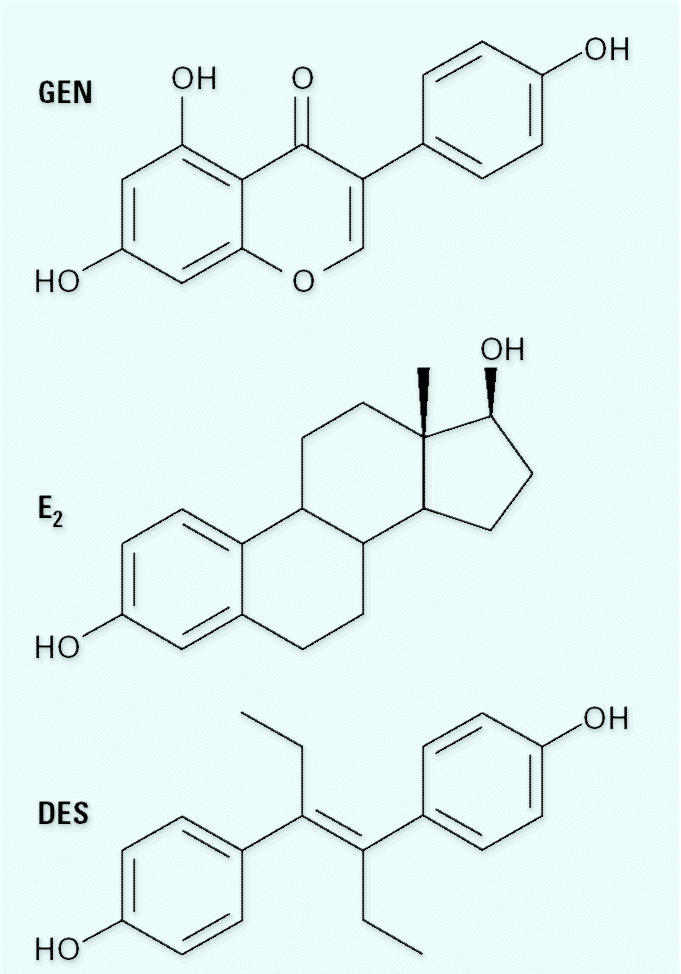
Chemical structure of GEN, E_2_, and DES.

**Figure 2:**
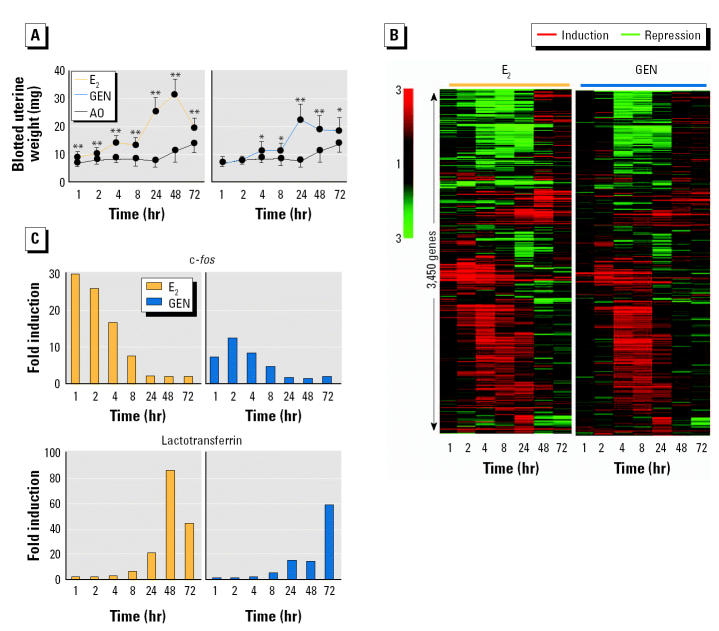
Induction of very similar multistage transcriptional responses in the mouse uterus by E_2_ and GEN. (*A*) Blotted uterine weights (mean ± SD) of sexually immature mice (*n* = 10/group) at different times after a single subcutaneous dose of E_2_ (400 μg/kg), GEN (250 mg/kg), or AO (control; 5 mL/kg). See text for details of experiments. (*B*) Temporal expression profiles of 3,450 genes up-regulated or repressed (> 1.5-fold) by either E_2_ (400 μg/kg) or GEN (250 mg/kg) at one or more of seven different time points. The magnitude of altered gene expression (fold change vs. time-matched vehicle control) is indicated by color; genes are grouped according to similarity of their temporal expression profiles (Pearson correlation-based hierarchical clustering). (*C*) Northern blot analysis of temporal expression pattern of early (c-*fos*) and late (lactotransferrin) estrogen-responsive genes; the fold induction of gene expression relative to time-matched vehicle controls was calculated after data were normalized to the expression of the control gene *RPB1* (accession number NM_009089).
*p < 0.05;
**p < 0.01; two-sided Student *t*-test.

**Figure 3:**
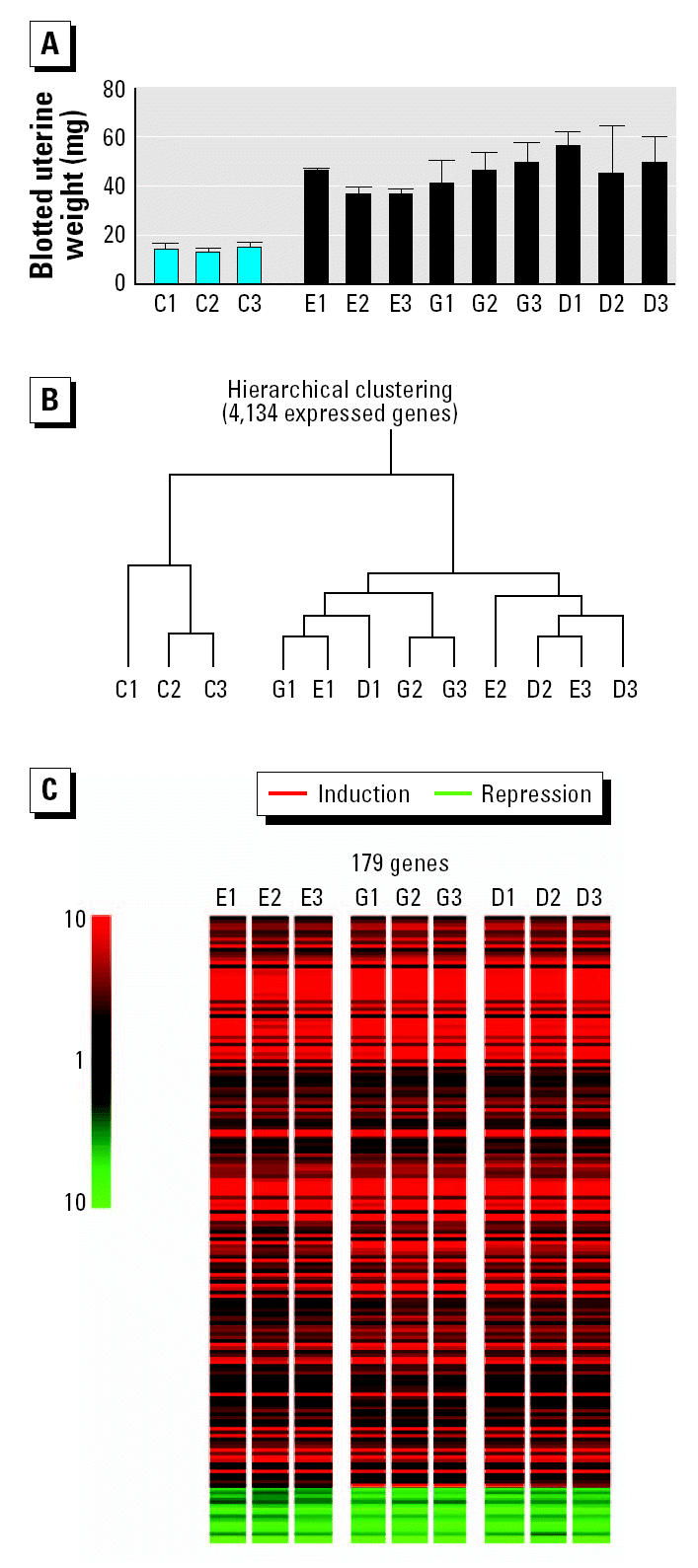
Equivalence of biologic responses induced in the mouse uterus by E_2_ (E), GEN (G), and DES (D). (*A*) Blotted uterine weights (mean ± SD) of three independent replicate groups (1–3) of sexually immature mice (*n* = 4/group) after three daily subcutaneous injections of either GEN (50 mg/kg), E_2_ (2.5 μg/kg), DES (2 μg/kg), or AO [control (C); 5 mL/kg]. (*B*) Unsupervised Euclidean-distance–based hierarchical clustering of 4,134 expressed genes. (*C*) Near-identical gene expression profiles induced by the three estrogens 72 hr after equipotent uterotrophic doses. Significant changes in gene expression induced by one or more of the three estrogens were identified by one-way ANOVA (parametric test, assuming equal variance). The magnitude of altered gene expression (fold change vs. vehicle control) is indicated by color.

**Table 1:** Blotted uterine weights and endometrial and epithelial cell heights (mean ± SD) after exposure to E_2_, GEN, or DES for 3 consecutive days.*^a^*

			Cell height (μm)	
Compound	Dose (per kg)	Blotted uterine weight (mg)	Endometrium	Epithelium
AO	5 mL	13.0 ± 2.4	159.0 ± 23.1 (11)	11.4 ± 1.1
E_2_	2.5 μg	45.3 ± 8.6*	246.1 ± 52.4* (9)	23.3 ± 1.4*
GEN	50 mg	39.8 ± 5.3*	273.7 ± 63.3* (12)	23.7 ± 3.1*
DES	2.0 μg	49.8 ± 13.0*	273.2 ± 55.9* (10)	22.6 ± 4.0*

There were 12 animals/group, but not all of the histopathology samples were suitable for analyses; numbers in parentheses indicate the number of animals per group from which the histology data were generated.

a Data were assessed for statistical significance using a two-sided Student *t*-test:

* *p* < 0.01.

**Table 2:** Quantitative data for 179 differentially expressed genes (from Figure 3C) regulated in the mouse uterus by all three estrogens (E_2_, GEN, and DES).*^a^*

		Fold change in expression (mean ± SD)		
Gene name	GenBank accession no.	E_2_	GEN	DES
**Up-regulated genes**				
Solute carrier family 9a3r1	U74079	1.8 ± 0.01	2.0 ± 0.1	2.0 ± 0.2
Keratin complex 2–8	X15662	2.6 ± 0.2	3.1 ± 0.2	3.1 ± 0.3
Laminin beta 3	U43298	4.3 ± 0.1	5.5 ± 1.1	5.3 ± 0.7
Claudin 7	AF087825	4.5 ± 0.5	6.5 ± 1.0	5.8 ± 0.6
bHLH-Zip transcription factor	U49507	2.6 ± 0.3	3.1 ± 0.3	2.9 ± 0.1
RIKEN cDNA 1200008D14	AW208938	3.0 ± 0.3	3.5 ± 0.1	3.3 ± 0.3
Basic HLH-domain containing, class B2	Y07836	5.9 ± 1.0	6.6 ± 0.9	6.6 ± 0.8
RIKEN cDNA 9930104H07	AW122310	3.0 ± 0.3	3.2 ± 0.4	3.3 ± 0.1
Fucosyltransferase 2	AF064792	27.5 ± 1.2	34.6 ± 8.5	36.7 ± 5.5
Deleted in polyposis 1	U28168	1.8 ± 0.1	2.0 ± 0.02	2.0 ± 0.1
Microsomal glutathione *S*-transferase 3	AI843448	2.9 ± 0.2	3.3 ± 0.6	3.3 ± 0.1
Tumor-associated Ca signal transducer 2	Y08830	4.0 ± 0.3	4.6 ± 0.9	4.6 ± 0.3
Calpain 5	Y10656	5.5 ± 0.4	6.3 ± 1.0	6.6 ± 0.6
Mitochondrial creatine kinase	Z13969	9.7 ± 1.1	12.2 ± 2.1	13.1 ± 1.8
ATPase 6v1a1	AW123765	2.0 ± 0.1	2.1 ± 0.2	2.1 ± 0.2
Tumor-associated Ca signal transducer 2	AI563854	8.0 ± 0.4	9.2 ± 1.0	8.5 ± 0.4
Lymphocyte antigen 6 complex, locus A	X04653	7.8 ± 0.9	8.8 ± 0.3	8.5 ± 0.4
Chloride channel calcium-activated 3	AV373378	26.4 ± 3.4	26.7 ± 1.0	26.2 ± 3.9
Small proline-rich protein 2I	AJ005567	23.9 ± 1.5	24.7 ± 1.3	23.6 ± 1.6
Oncoprotein induced transcript 1	AA615075	19.0 ± 3.1	20.0 ± 1.2	18.9 ± 2.5
Small proline-rich protein 2F	AJ005564	59.8 ± 8.4	65.8 ± 1.1	60.6 ± 2.6
Small proline-rich protein 2E	AJ005563	12.0 ± 1.0	12.9 ± 0.8	12.1 ± 0.9
Mucin 1	M84683	8.3 ± 0.6	8.6 ± 0.3	8.5 ± 0.5
Lipoocalin 2	X81627	150.3 ± 15.0	175.7 ± 10.5	162.8 ± 6.5
RIKEN cDNA 2210409B01	AF109906	3.5 ± 0.6	4.0 ± 0.3	3.8 ± 0.8
Interferon-activated gene 202A	M31418	7.9 ± 1.0	9.8 ± 2.5	8.8 ± 0.7
Nuclear ankyrin-repeat protein	AA614971	3.7 ± 0.6	4.3 ± 0.7	4.1 ± 0.9
RIKEN cDNA 5730469M10	AI850090	22.0 ± 5.8	30.5 ± 9.1	27.0 ± 6.5
RIKEN cDNA 1110034C02	AI837104	1.5 ± 0.1	1.6 ± 0.1	1.6 ± 0.03
IMAGE cDNA 4988271	AV373294	8.0 ± 2.5	10.6 ± 1.6	9.2 ± 1.1
RIKEN cDNA 5730493B19	AW122413	12.7 ± 0.3	19.0 ± 4.1	15.7 ± 0.9
Peptidoglycan recognition protein	AV092014	13.4 ± 1.5	18.3 ± 3.0	14.5 ± 2.1
Inhibin beta-B	X69620	13.6 ± 3.1	19.4 ± 4.7	16.0 ± 1.4
CEA-related cell adhesion molecule 2	AF101164	11.9 ± 1.6	17.8 ± 5.3	14.2 ± 1.7
Keratin complex 1–19	M36120	4.4 ± 0.4	5.5 ± 1.1	4.8 ± 0.5
CEA-related cell adhesion molecule 1	M77196	15.9 ± 2.4	23.9 ± 5.9	19.0 ± 3.8
SRC family-associated phosphoprotein 2	AB014485	2.7 ± 0.04	3.2 ± 0.4	2.9 ± 0.3
Peptidoglycan recognition protein	AF076482	7.7 ± 1.9	10.4 ± 2.7	9.0 ± 2.0
CEA-related cell adhesion molecule 1	M77196	19.5 ± 3.9	30.4 ± 8.8	22.2 ± 2.7
CEA-related cell adhesion molecule 1	X67279	6.4 ± 0.7	8.4 ± 1.1	7.1 ± 1.1
Spermidine N1-acetyl transferase	L10244	8.3 ± 0.9	11.2 ± 0.8	9.3 ± 0.6
RIKEN cDNA 0610007O07	AI851762	2.7 ± 0.1	3.0 ± 0.3	2.8 ± 0.1
Arginase 1	U51805	79.4 ± 9.8	131.9 ± 20.0	99.6 ± 14.5
Acetyl-coenzyme A synthetase 2	AW125884	2.2 ± 0.2	2.0 ± 0.1	2.2 ± 0.2
*v-erb*-b2 homolog 3	AI006228	3.4 ± 0.4	3.1 ± 0.6	3.4 ± 0.4
Phospholipase D3	AF026124	2.6 ± 0.2	2.4 ± 0.2	2.6 ± 0.2
RIKEN cDNA 0610031J06	AW122935	1.9 ± 0.1	1.8 ± 0.1	1.8 ± 0.1
Complement component 1q	X58861	2.1 ± 0.1	2.0 ± 0.1	2.0 ± 0.2
Scotin	AW123754	2.0 ± 0.1	2.0 ± 0.2	2.0 ± 0.2
CD24a antigen	M58661	3.2 ± 0.1	3.1 ± 0.1	3.3 ± 0.3
Argininosuccinate synthetase 1	M31690	2.7 ± 0.3	2.7 ± 0.3	2.8 ± 0.4
ATPase 6v1a1	U13837	2.1 ± 0.1	2.1 ± 0.2	2.2 ± 0.2
Gelsolin-like actin-capping protein	X54511	3.6 ± 0.5	3.7 ± 0.5	3.7 ± 0.2
Golgi phosphoprotein 2	AW125446	4.5 ± 0.5	4.6 ± 0.5	4.7 ± 0.1
Aldolase 1A	Y00516	2.3 ± 0.2	2.3 ± 0.1	2.4 ± 0.1
Cathepsin L	X06086	6.4 ± 0.8	6.3 ± 1.1	6.9 ± 0.4
CD14 antigen	X13333	3.0 ± 0.1	2.8 ± 0.1	3.1 ± 0.2
Decay accelerating factor 2	L41365	4.0 ± 0.1	3.8 ± 0.8	3.8 ± 0.2
Actin-related protein 2/3 complex 1B	AW212775	2.1 ± 0.2	2.1 ± 0.1	2.1 ± 0.2
Protective protein for β-galactosidase	J05261	2.0 ± 0.1	2.0± 0.1	2.0 ± 0.1
Elastase 1	M27347	2.7 ± 0.1	2.5 ± 0.2	2.6 ± 0.1
Connexin 26	M81445	10.6 ± 1.0	9.8 ± 0.5	10.4 ± 0.8
Ceruloplasmin	U49430	15.1 ± 2.8	15.0 ± 5.5	14.5 ± 2.1
Cathepsin H	U06119	3.0 ± 0.2	3.0 ± 0.3	3.0 ± 0.3
Basigin	Y16258	1.6 ± 0.1	1.5 ± 0.1	1.7 ± 0.1
Peptidylprolyl isomerase C–associated	X67809	2.2 ± 0.2	2.2 ± 0.1	2.4 ± 0.3
Glutathione reductase 1	AI851983	2.3 ± 0.2	2.3 ± 0.1	2.6 ± 0.3
START domain–containing 3	X82457	1.5 ± 0.1	1.4 ± 0.03	1.5 ± 0.01
CD68 antigen	X68273	4.6 ± 0.6	4.2 ± 0.6	5.0 ± 0.7
RIKEN cDNA E030027H19	AW211760	2.7 ± 0.3	2.7 ± 0.2	2.9 ± 0.1
cDNA sequence BC004044	AI461767	3.1 ± 0.2	3.4 ± 0.1	3.8 ± 0.5
E74-like factor 3	AF016294	5.1 ± 0.8	5.9 ± 0.5	6.5 ± 0.5
Glutathione *S*-transferase omega 1	AI843119	5.0 ± 1.1	4.6 ± 0.6	4.1 ± 0.7
Interferon-stimulated protein 20	AW122677	4.2 ± 0.1	4.3 ± 0.7	3.4 ± 0.4
Clusterin	D14077	3.6 ± 0.7	3.9 ± 0.9	3.4 ± 0.4
Galectin 3	X16834	7.4 ± 1.3	8.7 ± 0.7	6.9 ± 0.4
Small proline-rich protein 2A*^b^*	AJ005559	51.1 ± 4.0	78.3 ± 15.7	44.2 ± 5.2
Complement component 3*^b^*	K02782	14.8 ± 1.8	18.8 ± 1.0	14.8 ± 0.8
Small proline-rich protein 2C	AJ005561	220.3± 31.0	340.5 ± 37.1	214.1 ± 41.1
Small proline-rich protein 2G	AJ005565	9.4 ± 0.9	11.0 ± 0.3	9.6 ± 0.6
Prominin	AF039663	3.5 ± 0.5	3.6 ± 0.6	3.4 ± 0.3
Lactotransferrin*^b^*	J03298	88.7 ± 18.4	99.2 ± 13.9	76.9 ± 21.8
Carbonic anhydrase 2	M25944	7.9 ± 0.5	8.2 ± 0.9	7.3 ± 0.4
Complement component factor I	U47810	36.5 ± 4.4	38.4 ± 5.6	32.9 ± 4.2
Mannosidase 2alphaB1	U87240	2.0 ± 0.2	2.0 ± 0.1	1.9 ± 0.1
Small proline-rich protein 2B	AJ005560	32.9 ± 3.7	39.2 ± 1.9	30.7 ± 5.0
Small proline-rich protein 2A*^b^*	AJ005559	269.8 ± 23.7	329.1 ± 42.9	59.1 ± 40.8
RIKEN cDNA 5830413E08	AI849939	3.3 ± 0.4	3.3 ± 0.5	3.0 ± 0.3
RIKEN cDNA 1110029F20	AW125508	4.1 ± 0.1	4.1 ± 0.4	3.7 ± 0.1
Annexin A3	AJ001633	2.7 ± 0.5	4.2 ± 0.7	3.2 ± 0.6
Peptidase 4	U51014	2.0 ± 0.1	2.9 ± 0.3	2.3 ± 0.2
Laminin gamma 2	U43327	6.3 ± 1.3	17.3 ± 6.8	10.2 ± 1.0
Ubiquitin-like 3	AW120725	1.5 ± 0.1	1.8 ± 0.1	1.7 ± 0.03
Urate oxidase	M27695	23.8 ± 9.5	143.9 ± 62.9	43.8 ±17.7
Amiloride binding protein 1	AI197481	3.5 ± 1.0	10.1 ± 0.8	6.0 ± 1.2
Keratin complex 1–19	AU040563	4.5 ± 1.0	7.2 ± 1.0	5.6 ± 0.4
Activated leukocyte cell adhesion molecule	L25274	3.6 ± 0.8	5.1 ± 0.9	4.4 ± 0.5
CCAAT/enhancer binding protein β	M61007	2.3 ± 0.1	2.8 ± 0.3	2.6 ± 0.1
Peptidyl arginine deiminase, type I	AB013848	8.6 ± 0.7	15.8 ± 3.1	12.0 ± 1.7
Enolase 1 α	AI841389	2.5 ± 0.3	3.2 ± 0.5	3.0 ± 0.3
*p53* apoptosis effector related to *Pmp22*	AI854029	2.9 ± 0.3	4.1 ± 0.8	3.7 ± 0.5
β-Glucuronidase	M19279	1.9 ± 0.1	2.3 ± 0.2	2.2 ± 0.1
Leucine-rich α-2-glycoprotein	AW230891	9.3 ± 1.1	17.6 ± 4.1	14.5 ± 2.6
Quiescin Q6	AW123556	3.7 ± 0.2	5.5 ± 1.2	4.8 ± 0.7
GADD45a	U00937	1.9 ± 0.2	2.6 ± 0.3	2.3 ± 0.1
Alkaline phosphatase 2	J02980	9.2 ± 0.4	22.6 ± 6.2	15.8 ± 2.0
Immediate early response 3	X67644	5.5 ± 0.8	10.8 ± 2.2	8.9 ± 2.0
Progressive ankylosis	AW049351	2.2 ± 0.1	2.9 ± 0.4	2.8 ± 0.4
RAS p21 protein activator 4	AA163960	6.8 ± 0.9	14.2 ± 2.5	12.1 ± 1.7
Tumor-associated calcium signal transducer 1	M76124	2.1 ± 0.2	2.7 ± 0.3	2.6 ± 0.2
Hydroxysteroid (17-beta) dehydrogenase 11	AA822174	1.9 ± 0.1	2.3 ± 0.3	2.4 ± 0.1
Platelet-activating factor acetylhydrolase 1ba1	U57746	1.9 ± 0.1	2.2 ± 0.2	2.3 ± 0.1
Branched chain aminotransferase 1	U42443	2.4 ± 0.2	3.4 ± 0.2	3.4 ± 0.01
RIKEN cDNA 2400004E04	AI846720	1.7 ± 0.1	2.4 ± 0.2	2.3 ± 0.2
Myeloblastosis oncogene	M12848	2.8 ± 0.4	5.6 ± 1.1	5.0 ± 0.2
K^+^ conductance calcium-activated channel N4	AF042487	3.1 ± 0.2	4.2 ± 0.6	3.5 ± 0.8
ATPase 6v1b2	AI843029	1.7 ± 0.1	1.8 ± 0.1	1.8 ± 0.1
Cystic fibrosis transmembrane regulator	M60493	3.4 ± 0.5	4.5 ± 0.8	3.9 ± 0.3
RIKEN cDNA 1110008P14	AI839839	4.3 ± 0.4	6.0 ± 1.0	5.1 ± 0.2
Fused toes	Z67963	2.6 ± 0.2	3.2 ± 0.3	2.8 ± 0.1
Solute carrier family 39a8	AW124340	3.5 ± 0.5	4.5 ± 0.7	3.8 ± 0.3
Cytochrome b-561	AI846517	2.2 ± 0.2	2.5 ± 0.2	2.3 ± 0.2
Secreted phosphoprotein 1	X13986	30.2 ± 3.3	47.4 ± 7.4	31.1 ± 2.7
Ion transport regulator Fxyd3	X93038	5.3 ± 0.4	6.8 ± 1.1	5.5 ± 0.6
Janus kinase 3	L40172	2.1 ± 0.2	2.5 ± 0.2	2.2 ± 0.2
Cytochrome b-245alpha	AW046124	2.9 ± 0.4	3.6 ± 0.4	2.9 ± 0.2
RIKEN cDNA A430096B05	AI465965	6.3 ± 1.0	8.6 ± 0.03	6.3 ± 0.6
Small proline-rich protein 2J	AJ005568	8.6 ± 2.1	13.8 ± 3.2	8.5 ± 0.7
Cathepsin B	M65270	2.2 ± 0.1	2.6 ± 0.2	2.2 ± 0.1
RIKEN cDNA 1600025H15	AI842734	2.2 ± 0.1	2.7 ± 0.3	2.2 ± 0.2
c-*fos* oncogene*^b^*	V00727	3.2 ± 0.4	4.7 ± 0.9	3.7 ± 0.8
Guanine nucleotide binding protein γ5	AI843937	1.6 ± 0.1	1.8 ± 0.1	1.6 ± 0.03
Serine palmitoyltransferase lc2	U27455	1.6 ± 0.1	2.0 ± 0.2	1.7 ± 0.1
Cystatin B	U59807	1.5 ± 0.1	1.7 ± 0.1	1.5 ± 0.02
Villin 2	X60671	1.9 ± 0.2	2.4 ± 0.3	1.9 ± 01
RIKEN cDNA 0610010O12	AI849011	1.9 ± 0.1	2.5 ± 0.3	1.9 ± 0.03
Matrix metalloproteinase 7	L36244	47.8 ± 18.6	208.6 ± 83.3	48.2 ± 11.9
RIKEN cDNA 4930422J18	AV376312	2.0 ± 0.3	2.9 ± 0.3	2.0 ± 0.2
RIKEN cDNA 1700017B05	AW049360	1.6 ± 0.1	2.0 ± 0.1	1.6 ± 0.1
Galactosidase beta 1	M57734	1.8 ± 0.1	2.0 ± 0.1	1.7 ± 0.1
Cathepsin C	U74683	2.6 ± 0.1	3.3 ± 0.2	2.3 ± 0.3
Interferon-stimulated protein 15	X56602	3.6 ± 0.6	2.0 ± 0.2	3.8 ± 0.7
MAP kinase–interacting kinase 2	Y11092	1.8 ± 0.1	1.4 ± 0.1	1.9 ± 0.1
Glutathione *S*-transferase theta 2	X98056	3.5 ± 0.4	2.9 ± 0.4	3.6 ± 0.2
Gene name	accession no.	E_2_	GEN	DES
Homeobox B6	M18401	1.5 ± 0.02	1.5 ± 0.1	1.6 ± 0.03
Procollagen VIalpha 3	AF064749	2.1 ± 0.2	1.9 ± 0.2	2.1 ± 0.02
Interferon regulatory factor 7	U73037	11.7 ± 0.9	8.1 ± 0.7	12.6 ± 1.5
Scavenger receptor class B2	AB008553	2.7 ± 0.1	2.4 ± 0.3	2.6 ± 0.2
Polyimmunoglobulin receptor	AB001489	8.1 ± 0.7	6.2 ± 0.8	7.9 ± 1.0
Proteasome subunit β10	Y10875	2.1 ± 0.04	1.9 ± 0.04	2.1 ± 0.1
RIKEN cDNA 0610010E05	AV312736	2.9 ± 0.3	2.5 ± 0.2	2.7 ± 0.4
RIKEN cDNA 0610010E05	AI854839	3.7 ± 0.5	3.0 ± 0.1	3.4 ± 0.3
Xanthine dehydrogenase	X75129	12.2 ± 2.2	8.9 ± 1.2	10.1 ± 1.5
Prominin	AF039663	3.5 ± 0.2	2.9 ± 0.2	3.1 ± 0.3
Interferon-induced protein IFIT1	U43084	17.5 ± 2.9	9.9 ± 1.5	14.0 ± 2.1
Interferon-induced protein IFIT3	U43086	8.1 ± 2.3	4.7 ± 0.2	6.7 ± 0.6
Proteasome subunit β8	U22033	2.0 ± 0.1	1.8 ± 0.2	2.1 ± 0.1
RIKEN cDNA 1600023A02	AW121336	1.9 ± 0.1	1.7 ± 0.1	2.0 ± 0.04
Small proline-rich protein 1A	AF057156	11.1 ± 3.7	8.6 ± 0.8	14.7 ± 0.8
MAP kinase-interacting kinase 2	AI845732	2.0 ± 0.1	1.7 ± 0.1	2.0 ± 0.2
Lymphocyte antigen 6 complex, locus E	U47737	2.0 ± 0.01	1.7 ± 0.1	2.0 ± 0.1
Guanylate nucleotide binding protein 2	AJ007970	3.0 ± 0.1	2.0 ± 0.2	2.6 ± 0.1
Peptidyl arginine deiminase, type II*^b^*	D16580	1.9 ± 0.3	9.1 ± 0.3	5.2 ± 1.5
**Down-regulated genes**				
Solute carrier family 29a1	AI838274	2.0 ± 0.2	2.9 ± 0.3	2.5 ± 0.1
Lymphocyte specific 1	D49691	1.6 ± 0.1	2.3 ± 0.2	1.8 ± 0.1
Claudin 5	U82758	2.0 ± 0.2	2.8 ± 0.1	2.7 ± 0.5
Potassium channel td12	AI842065	1.6 ± 0.04	2.0 ± 0.04	2.1 ± 0.1
Zinc finger homeobox 1a	D76432	1.5 ± 0.1	1.7 ± 0.1	1.8 ± 0.01
Monoamine oxidase A	AI848045	2.3 ± 0.2	2.7 ± 0.2	2.5 ± 0.4
Histidine decarboxylase	X57437	4.8 ± 0.8	7.1 ± 0.6	5.4 ± 0.8
α-2 Adrenergic receptor	M97516	3.0 ± 0.3	4.2 ± 1.1	3.6 ± 0.3
Transcription factor 21	AF035717	1.8 ± 0.2	2.2 ± 0.2	2.0 ± 0.1
Homeobox D8	X56561	2.2 ± 0.1	2.8 ± 0.03	2.5 ± 0.2
Carboxypeptidase X2	AF017639	4.1 ± 0.7	5.2 ± 1.0	4.2 ± 0.2
RIKEN cDNA A230106A15	AI848841	3.8 ± 0.2	4.7 ± 0.5	4.2 ± 0.7
Reduced expression 3	AA790008	3.1 ± 0.2	3.5 ± 0.3	3.2 ± 0.4
TGF-β binding protein 4	AA838868	1.8 ± 0.1	2.1 ± 0.1	1.8 ± 0.2
Keratoepithelin*^b^*	L19932	11.5 ± 2.5	12.6 ± 0.9	9.7 ± 3.6
GLI-Kruppel family member GLI	AB025922	11.6 ± 0.8	12.2 ± 2.6	8.2 ± 2.6

Abbreviations: CEA, carcinoembrionary antigen; SRC, steroid receptor coactivator; TGF, transforming growth factor.

a Gene names (derived from the NetAffx database; Liu et al. 2003), GenBank accession numbers (GenBank 2004), and mean (± SD) fold induction/repression of gene expression are shown in the same order as the gene cluster in Figure 3C.

b Genes mentioned in the text.
